# Aurora Kinase A is a Biomarker for Bladder Cancer Detection and Contributes to its Aggressive Behavior

**DOI:** 10.1038/srep40714

**Published:** 2017-01-19

**Authors:** Aaron Mobley, Shizhen Zhang, Jolanta Bondaruk, Yan Wang, Tadeusz Majewski, Nancy P. Caraway, Li Huang, Einav Shoshan, Guermarie Velazquez-Torres, Giovanni Nitti, Sangkyou Lee, June Goo Lee, Enrique Fuentes-Mattei, Daniel Willis, Li Zhang, Charles C. Guo, Hui Yao, Keith Baggerly, Yair Lotan, Seth P. Lerner, Colin Dinney, David McConkey, Menashe Bar-Eli, Bogdan Czerniak

**Affiliations:** 1 Department of Cancer Biology, The University of Texas at MD Anderson Cancer Center, 1515 Holcombe Blvd., Houston, Texas 77030, USA; 2 Department Pathology, The University of Texas at MD Anderson Cancer Center, 1515 Holcombe Blvd., Houston, Texas 77030, USA; 3 Department Urology, The University of Texas at MD Anderson Cancer Center, 1515 Holcombe Blvd., Houston, Texas 77030, USA; 4 Department Bioinformatics & Comp Biology, The University of Texas at MD Anderson Cancer Center, 1515 Holcombe Blvd., Houston, Texas 77030, USA; 5 Department of Urology, UT Southwestern University, Dallas, TX 75390, USA; 6 Scott Department of Urology, Baylor College of Medicine, Houston, TS 77030, USA

## Abstract

The effects of AURKA overexpression associated with poor clinical outcomes have been attributed to increased cell cycle progression and the development of genomic instability with aneuploidy. We used RNA interference to examine the effects of AURKA overexpression in human bladder cancer cells. Knockdown had minimal effects on cell proliferation but blocked tumor cell invasion. Whole genome mRNA expression profiling identified nicotinamide N-methyltransferase (NNMT) as a downstream target that was repressed by AURKA. Chromatin immunoprecipitation and NNMT promoter luciferase assays revealed that AURKA’s effects on NNMT were caused by PAX3-mediated transcriptional repression and overexpression of NNMT blocked tumor cell invasion *in vitro*. Overexpression of AURKA and activation of its downstream pathway was enriched in the basal subtype in primary human tumors and was associated with poor clinical outcomes. We also show that the FISH test for the AURKA gene copy number in urine yielded a specificity of 79.7% (95% confidence interval [CI] = 74.2% to 84.1%), and a sensitivity of 79.6% (95% CI = 74.2% to 84.1%) with an AUC of 0.901 (95% CI = 0.872 to 0.928; P < 0.001). These results implicate AURKA as an effective biomarker for bladder cancer detection as well as therapeutic target especially for its basal type.

Although a majority of patients present with superficial bladder cancer, its progression to muscle invasion identifies a potentially lethal form of disease as approximately half of these patients will die of metastatic spread^1^,^2^. The management of invasive bladder cancer has not changed in over 3 decades and still relies on definitive surgery with or without perioperative cisplatin-based chemotherapy^3^. Unfortunately, the overall impact of chemotherapy on survival is very modest^4^, and it is currently impossible to distinguish patients with organ-confined disease who will be cured with surgery alone from those with subclinical metastases at presentation. Defining the molecular mechanisms that underlie bladder cancer invasion and metastasis could identify biomarkers predicting the presence of metastases and therapeutic targets that could be used to block the progression of the disease, dramatically altering the outcome.

Aurora kinase A (AURKA) is a member of a family of serine/threonine kinases that plays a well-established role in promoting cell cycle entry and assembly of the mitotic spindle, thereby facilitating mitosis^5^,^6^,^7^. Overexpression of AURKA is observed in a variety of different cancers^8^,^9^,^10^,^11^, resulting in defective control of the mitotic spindle checkpoint leading to aneuploidy, and it has been an attractive therapeutic target for over a decade^12^,^13^,^14^,^15^,^16^. Amplification of AURKA is observed in invasive bladder cancer and is associated with poor clinical outcomes that have been attributed to AURKA effects on genomic instability and mitosis^17^,^18^. An in-depth functional characterization of AURKA’s biological effects has not been performed to date, so this conclusion is largely an assumption.

Here we use human bladder cancer as a model disease and show that AURKA contributes to bladder cancer aggressiveness by enhancing the invasiveness of tumor cells through modulation of the transcription factor Pax-3 causing the downregulation of the nicotinamide N-methyltrasferase (NNMT) gene resulting in the downstream overexpression of matrix metalloproteinases (MMP). These preclinical observations were validated on several non-overlapping cohorts comprised of 693 human bladder cancers using tissue microarray and two genomically profiled sample sets which showed that there is a unique subset of bladder cancers with overexpression of AURKA and downregulation of NNMT characterized by aggressive behavior. The overexpression of AURKA combined with the expression signature of AURKA downstream regulatory genes was enriched in the basal intrinsic molecular subtype of bladder cancer which exhibited highly aggressive clinical behavior. Finally, we show that the AURKA gene copy number could be used as an effective biomarker for the non-invasive detection of bladder cancer. These studies were conducted on a multi-institutional cohort, comprising 711 human subjects, and they showed that flouresence *in situ* hybridization (FISH) for AURKA copy number performed on cells from voided urine, can be used for the non-invasive detection and monitoring of bladder cancer.

## Results

### AURKA controls invasion in bladder cancer cells

**Figure 1:**
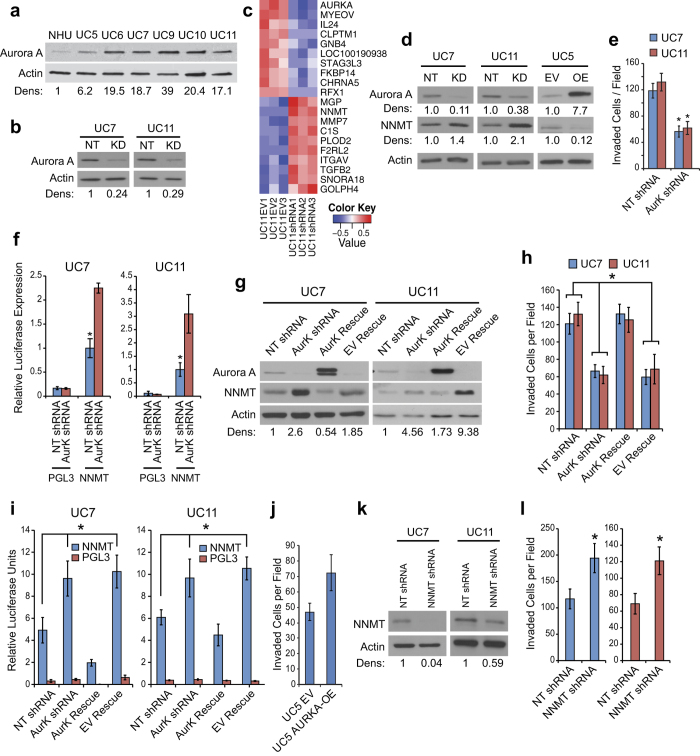
Effects of AURKA A on NNMT expression and cell invasion. (**a**) Panel of bladder cancer cells shows different levels of AURKA expression in UC5, UC6, UC7, UC 9, UC10 and UC11 cell lines. Note low level of AURKA expression in cultured normal urothelial (NHU) cells. Full length blots are shown in Supplementary Fig. S6. (**b**) Using a lentiviral shRNA construct AURKA was silenced by ~75% in UC7 and UC11 cells. Full length blots are shown in Supplementary Fig. S6. (**c**) Heatmap of most significantly up- and down-regulated genes after silencing of AURKA in UC11 cell line. (**d**) AURKA was silenced using a lentiviral vector. Upon silencing, NNMT is upregulated by 3–4 fold in UC7 and UC11 cell lines. In contrast, overexpression of AURKA in UC5 cells resulted with downregulation of NNMT. Full length blots are shown in Supplementary Fig. S6. (**e**) Upon silencing of AURKA cell invasion is decreased by ~3 fold in both UC7 and UC11 cells in a matrigel invasion assay (*P < 0.01). (**f**) A luciferase promoter analysis of NNMT reveals that NNMT’s upregulation is transcriptionally regulated in both cell lines upon silencing of AURKA (*P < 0.05). (**g**) AURKA KO UC7 and UC11 cells were treated with AURKA rescue vectors and western blot was performed to verify the rescue of AURKA and NNMT expression. Full length blots are shown in Supplementary Fig. S6. (**h**) Quantitation of cell invasion through matrigel coated invasion chambers in AURKA silenced and rescued UC7 and UC11 cell lines (*P < 0.05). (**i**) Luciferase based promoter analysis of the NNMT promoter indicated that when AURKA is rescued, luciferase expression is returned to lower than normal levels in both UC7 and UC11cell lines (*P < 0.05). (**j**) Overexpression of AURKA in UC5 cells which resulted in downregulation of NNMT (see Fig. 1d) increased cell invasion in matrigel coated chambers. (**k**) NNMT was silenced 80–90% in UC7 and UC11 cell lines using lentiviral shRNA directed against NNMT. A reduction of NNMT expression was observed by western blot. Full length blots are shown in Supplementary Fig. S6. (**l**) An increase in cell invasion was observed in both cell lines in NNMT shRNA treated cells when compared to NT shRNA (*P < 0.05).

In a previous study, we demonstrated that AURKA mRNA levels varied widely in a panel of human bladder cancer cell lines^19^. We selected six cell lines showing a spectrum of expression levels of AURKA and used immortalized normal human urothelial cells (NHU) which expressed a minimal level of AURKA as a reference. AURKA expression levels varied from six times higher in UC5 cells to 39 times higher in UC9 cells when compared to NHU cells (Fig. 1a). We selected two of these lines (UC7 and UC11) expressing 18 and 17 times higher level of AURKA when compared to NHU cells for more in-depth functional analyses. In order to evaluate the biologic effects of high-level AURKA expression, we transduced the UC7 and UC11 cells with an AURKA-specific shRNA construct or a non-targeting control and characterized the effects on proliferation and other biological properties associated with tumor progression. Silencing resulted in at least a 75% reduction in AURKA protein expression in both cell lines (Fig. 1b). To better understand the molecular mechanisms controlled by AURKA, we first analyzed the consequences of AURKA knockdown and rescue on global mRNA expression. Overall, the number of genes affected by AURKA silencing was fairly small (Fig. 1c; Supplementary Table S1). However, AURKA modulation had strong effects on the expression of NNMT, which was up-regulated by AURKA knockdown and reduced by AURKA rescue in both cell lines (Fig. 1d). In contrast, overexpression of AURKA in UC5 cells (low expression of AURKA) resulted with down-regulation of NNMT (Fig. 1d) Since NNMT is implicated in invasion and metastases, these results directed our attention to the potential role of AURKA in tumor progression. In fact, silencing of AURKA had no effect on proliferation (Supplementary Fig. S1) but reduced cell invasion by ~3 fold in both lines as measured by modified Boyden chamber assay (Fig. 1e).

To determine whether AURKA knockdown affected NNMT transcription, we transfected the AURKA-modulated cells with a luciferase-based NNMT promoter reporter construct. AURKA silencing increased NNMT promoter activity (Fig. 1f), while the shRNA-resistant AURKA expression construct reversed the induction of NNMT promoter activity caused by knockdown (Fig. 1g) confirming that AURKA directly controlled NNMT transcription. The increased activity of NNMT promoter tested by luciferase assay and the *in vitro* suppression of invasiveness after the silencing of AURKA could be reversed by its rescued expression (Fig. 1h,i). Overexpression of AURKA in UC5 cells resulted in increased invasiveness of these cells (Fig. 1j). To further explore the effects of NNMT expression on invasion, we transduced the UC7 and UC11 cells with an NNMT-specific shRNA construct or non-targeting control vector (Fig. 1k). Knockdown resulted in a 2-fold increase in cell invasiveness in both lines (Fig. 1l).

**Figure 2:**
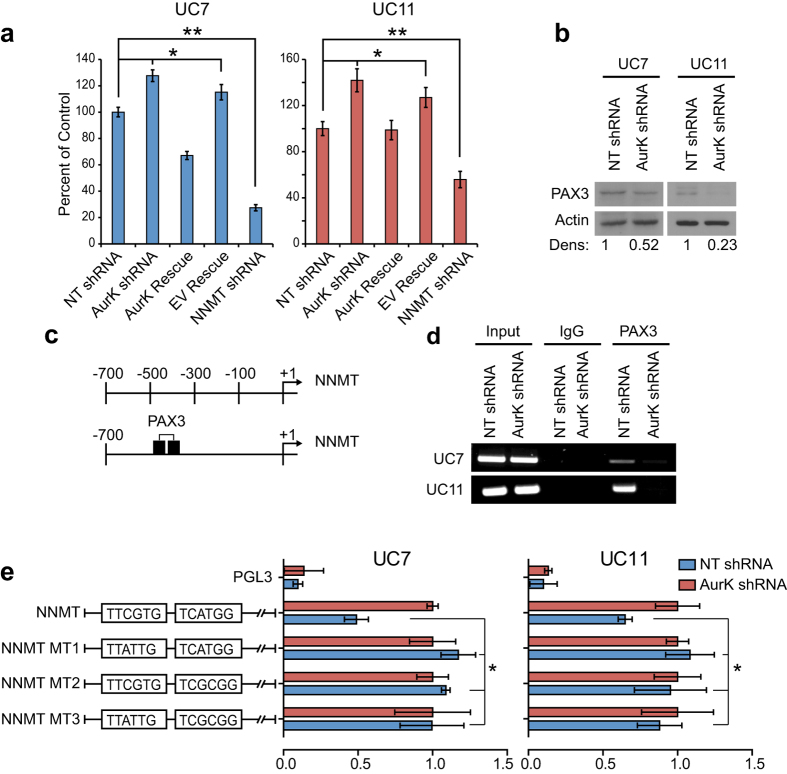
Transcriptional regulation of NNMT expression. (**a**) An NNMT activity assay was performed on UC7 and UC11 cell lines. The activity of NNMT normalized to actin correlated with its expression levels (*P < 0.05, **P < 0.01). (**b**) Western blotting revealed that expression levels of PAX3 decrease upon silencing of AURKA. Full length blots are shown in Supplementary Fig. S7. (**c**) Schematic presentation of the NNMT promoter. Two PAX3 binding sites are located within 500 bp upstream of the NNMT transcription start site. (**d**) Chromatin immunoprecipitation indicates that PAX3 binds to the NNMT promoter and represses its expression in both UC7 (top) and UC11 (bottom) cell lines. Full length gels are shown in Supplementary Fig. S7. (**e**) Luciferase based promoter analysis of the NNMT promoter with mutations in either of the PAX3 binding sites leads to an increase in luciferase expression in the non-targeted (NT) cells to similar levels as the AURKA KO cells. Mutation at both sites together did not have an additive effect, indicating that both sites are necessary for suppression of NNMT transcription (*P < 0.05).

Since the NNMT catalyzes the N-methylation of nicotinamide we analyzed the effects of AURKA modulation on NNMT’s enzymatic activity using liquid chromatography and quantified the methylation of nicotinamide (measured by accumulation of N-methynicotinamide (NMN) with ^14^C radiolabelled S-adenosyl-L-methionine (C-Ado-Met) as a methyl donor). UC7 and UC11 cells with silenced AURKA contained 30–50% higher levels of the NMN product while NNMT silenced cells contained 50–70% lower levels of NMN when compared to the controls. Rescued expression of AURKA in the AURKA-silenced cells restored NMN levels to those observed in the controls (Fig. 2a).

The NNMT promoter region spanning approximately 1Kb upstream of the transcription initiation site contains multiple potential transcription factor binding sites (STAT-3, HNF-1, CREB, ATF-1 and PAX3). We focused on the transcription factors that were modulated by AURKA silencing. Among several candidates, the expression levels of PAX3 decreased approximately 50% in both UC7 and UC11 cells after silencing of AURKA (Fig. 2b) and prior work demonstrated that PAX3 inhibited the transcription of NNMT^20^. To determine whether PAX3 directly affected the transcription of NNMT, we performed chromatin immunoprecipitation assays focused on two putative PAX3 binding sites (Fig. 2c). The results confirmed that PAX3 interacted with both of them. Silencing of AURKA reduced PAX3 binding to both sites in both cell lines (Fig. 2d). To further confirm the role of PAX3 in the transcriptional regulation of NNMT, the NNMT promoter was cloned into a PGL3 vector and the effects of mutations in both binding sites were evaluated in luciferase assays. Mutations in either binding site increased the activity of the promoter by over 50%. The presence of synchronous mutations in both sites did not have an additive effect, indicating that PAX3 binding to both sites is necessary for NNMT suppression (Fig. 2e).

**Figure 3:**
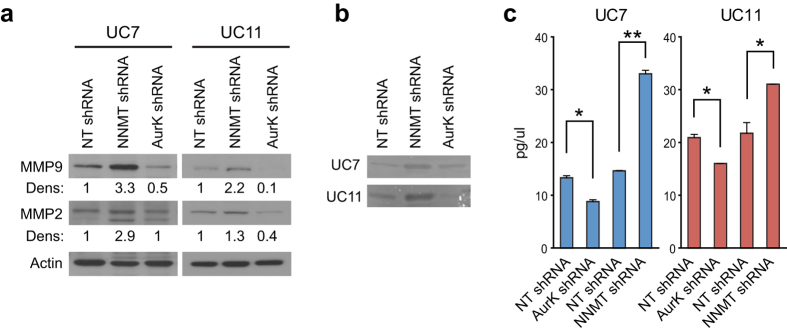
Effects of AURKA and NNMT on MMPs expression. (**a**) Western blots for MMP2 and MMP9 indicate an increase in both MMPs in NNMT KO cells and a decrease in both AURKA KO cells. Full length blots are shown in Supplementary Fig. S8. (**b**) Zymogram indicates increased levels of pro- and active forms of MMP2 in both UC7 and UC11 cell lines in NNMT KO cells, while a decrease in both pro- and active forms was observed in AURKA KO cells. Full length zymograms are shown in Supplementary Fig. S8. (**c**) MMP2 ELISA shows higher MMP2 levels in NNMT KO cells and lower levels in AURKA KO cells in both UC7 and UC11 cell lines (*P < 0.05, **P < 0.01).

Since matrix metalloproteinases (MMPs) play important roles in tumor invasion, and MMP2 and MMP9 have been shown to play roles in aggressive variants of bladder cancer^21^,^22^,^23^, we investigated whether modulation of AURKA and/or NNMT altered their expression. The silencing of AURKA decreased the expression of both MMPs, whereas the silencing of NNMT increased their expression levels (Fig. 3a). Accordingly, the silencing of AURKA decreased MMP activity while the silencing of NNMT increased MMP activity as shown by zymography assay (Fig. 3b) and by ELISA for MMP-2 expression (Fig. 3c).

### Overexpression of AURKA defines aggressive basal bladder cancer

**Figure 4:**
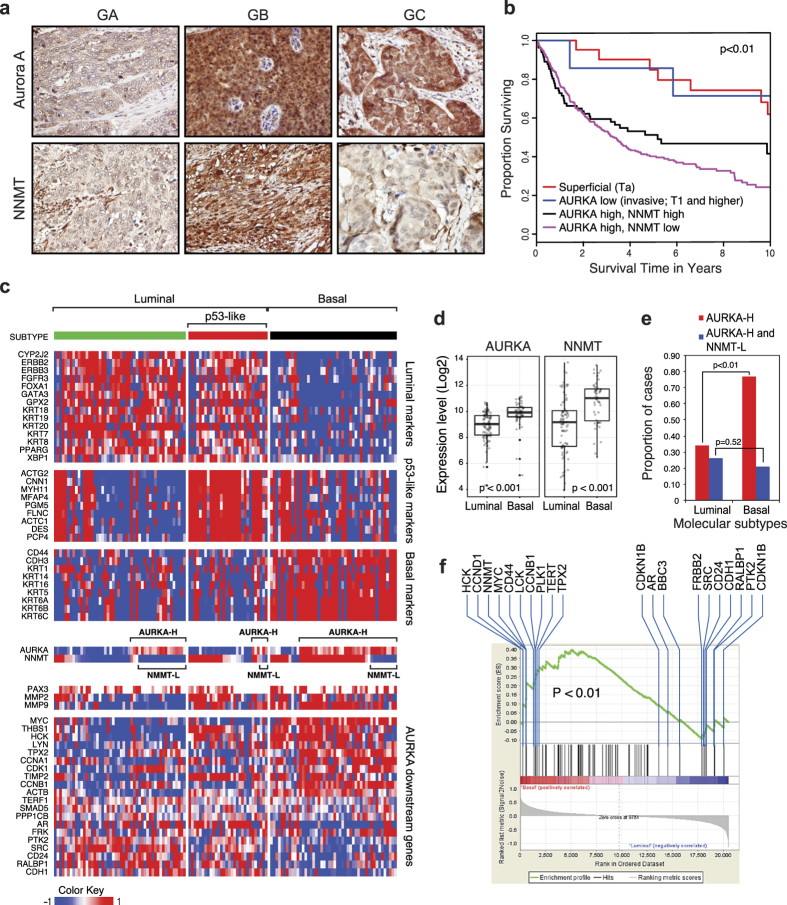
Overexpression of AURKA and down-regulation of NNMT defines aggressive variant of bladder cancer. (**a**) Expression levels of Aurora A and NNMT genes revealed by immunohistochemical staining in selected tumor samples from tissue microarray showing three paired patterns characteristic for three groups of invasive (T1 and higher) bladder cancer: GA (AURKA and NNMT with low expression), GB (AURKA and NNMT with high expression) and GC (AURKA with high expression and NNMT with low expression). (**b**) Kaplan-Meier analyses of disease-specific survivals in four groups of samples. Superficial (Ta) tumors were separated from invasive (T1 and higher). The invasive tumors were divided into three groups corresponding to samples with (1) low levels of AURKA expression (AURKA-L); (2) high levels of AURKA (AURKA-H), and high levels of NNMT (NNMT-H); and (3) high levels of AURKA (AURKA-H) and low levels of NNMT (NNMT-L). (**c**) Expression pattern of AURKA and AURKA signature genes in molecular subtypes of bladder cancer in the TCGA bladder cancer cohort comprising 128 high-grade muscle invasive bladder tumor samples which were classified into luminal, p53-like, and basal subtypes using the previously published algorithm. Subsets of samples with high levels of AURKA (AURKA-H) as well as subsets of samples showing overexpression of AURKA (AURKA-H) with downregulation of NNMT in molecular subtypes are shown. (**d**) Expression levels of AURKA and NNMT in luminal and basal subtypes of bladder cancer. (**e**) Proportions of cases with overexpression of AURKA (AURORA-H) and proportions of cases which show overexpression of AURKA (AURKA-H) with downregulation of NNMT (NNMT-L) in luminal and basal subtypes of bladder cancer. (**f**) Enrichment of AURKA downstream regulatory pathways in basal as compared to luminal subtypes of bladder cancer revealed by GSEA.

Together, our results demonstrated that AURKA and NNMT play antagonistic roles in the regulation of invasion in bladder cancer cells *in vitro*. To determine the relevance of these observations to human tumors, we measured the expression of AURKA and NNMT in 423 primary bladder cancers using immunohistochemistry and image analysis on tissue microarrays. In our analytical approach we first dichotomized the tumors into superficial (Ta) and invasive (T1 and higher). The results identified three distinct groups of invasive tumors, one with low levels of both AURKA and NNMT (comparable to those present in normal control urothelium), another with overexpression of both AURKA and NNMT, and a third group with overexpression of AURKA and low levels of NNMT (Fig. 4a; Supplementary Fig. S2). By comparing the clinical outcomes in the subsets, we observed that the cancers that expressed high levels of AURKA and low levels of NNMT had the worst outcomes and were associated with the shortest disease-specific survival (Fig. 4b). Interestingly, a subset of invasive tumors with low levels of AURKA expression showed favorable outcomes overlapping with those of superficial tumors.

In an attempt to validate these findings in independent cohorts and address whether AURKA played a role in specific molecular subsets of bladder cancer, we investigated the relationships between AURKA and NNMT expression levels and outcomes in two genomically characterized cohorts (The Cancer Genome Atlas, TCGA; The University of Texas MD Anderson Cancer Center, MDA) comprising 128 and 142 bladder samples, respectively. We classified the tumors according to recently identified intrinsic basal and luminal subtypes of bladder cancer (Fig. 4c; Supplementary Fig. S3a)^24^,^25^,^26^,^27^. In addition, a subset of luminal tumors was classified as p53-like^24^,^25^,^26^,^27^. In both of these cohorts, AURKA and NNMT expression levels were significantly higher in basal tumors when compared to luminal (Fig. 4d; Supplementary Fig. S3b). In the MDA cohorts, both subsets of invasive tumors (luminal and basal) showed the expression levels of AURKA and NNMT significantly higher when compared to superficial tumors (Supplementary Fig. S3b). In both TCGA and MDAnderson cohorts the proportion of AURKA overexpressing tumors was higher in the basal subtype (77% in TCGA and 67% in MDA) than in the luminal subtype (34% in TCGA and 46% in MDA). Both cohorts also contain subsets of cases with overexpression of AURKA and downregulation of NNMT. The TCGA cohort showed that 26% of luminal and 21% of basal cancers showed these characteristics (Fig. 4e). In the MD Anderson cohort 29% of luminal cancers showed overexpression of AURKA and downregulation of NNMT. In this cohort, only 4% of basal tumors showed this feature (Supplementary Fig. S3c). In general, basal tumors in both cohorts showed strong enrichment of the genes representing downstream regulatory targets of AURKA (Fig. 4f; Supplementary Fig. S3d). Basal tumors were associated with shorter disease specific survival in the MDA cohort where mature clinical follow-up data were available (Supplementary Fig. S3e).

Genomic instability, as measured by copy number variations, was actually higher in the luminal tumors within the TCGA cohort (Supplementary Fig. S4a–c), consistent with the idea that AURKA’s biological effects may be more tightly linked to invasion and metastasis rather than to genomic instability. Moreover, AURKA expression level could not be correlated with the genomic instability as measured by the fraction of genome with copy number variations (Supplementary Fig. S4d,e). These results are in agreement with the fact that the MD Anderson luminal tumors overlap significantly with the “genomically unstable” subtype identified by a group at Lund University^28^,^29^.

### AURKA FISH study in voided urine

**Figure 5:**
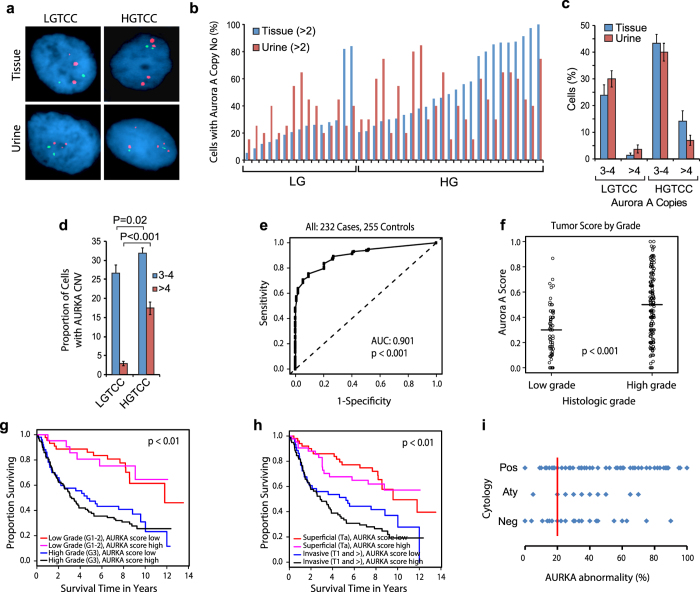
Detection of bladder cancer cells in tissue and voided urine by FISH with a probe specific for AURKA. (**a**) Dual-fluorescence FISH test with probes for AURKA (red) and the chromosome 20 α-satellite DNA (green) performed on 2 pairs of tissue and urine from the same patient with LGTCC and HGTCC respectively. Nuclei were counterstained with DAPI (blue). (**b**) Quantitative FISH analysis of AURKA gene copy numbers in tissue and voided urine specimens from 20 patients. The percentage of abnormal cells with more than 2 copies of AURKA in the individual patients is shown. (**c**) Mean percentage of cells with 3–4 copies and more than 4 copies of AURKA in matching tissue and urine samples from patients with LGTCC and HGTCC. (**d**) Average proportion of cells in voided urine showing 3–4 or more than 4 copies of AURKA in LGTCC and HGTCC detected in voided urine. (**e**) Receiver operating characteristic (ROC) curve for the set consisting of 232 urine samples from patients with bladder cancer and 255 urine samples from control subjects (126 healthy controls and 129 individuals with benign non-neoplastic disorders of the urinary tract). The AURKA FISH test for the detection of bladder cancer showed an area under the receiver operating characteristic curve (AUC) of 0.895 (95% confidence interval [CI] = 0.984 to 1.000). (**f**) AURKA gene FISH score in voided urine by histological grade of TCC stratified into low and high groups. Horizontal bars designate the mean AURKA gene score for TCCs of low and high histological grade. P value was calculated from two-sided Mann–Whitney *t* test. (**g**) Kaplan-Meier analyses of overall survival in low-grade (G1-2) and high grade (G3) tumors according to AURKA score. (**h**) Kaplan-Meier analyses of overall survival in superficial (Ta) and invasive (T1 and >) tumors according to AURKA score. (**i**) Comparison of AURKA FISH test results with cytologic analyses of voided urine in 144 samples from patients with bladder cancer.

Our previous studies have shown that AURKA is frequently amplified and overexpressed in bladder cancer^17^,^19^. We have also shown that a fluorescence *in situ* hybridization (FISH) test for AURKA copy number in cells from voided urine could be used to detect bladder cancer and that the quantitative assessment of AURKA copy number provides an estimate of bladder cancer aggressiveness^19^. Here we validated the test on a large multi-institutional cohort of voided urine samples from 487 subjects. We initially tested the AURKA FISH probe on paired samples of voided urine and bladder tumor tissue from a cohort of 40 patients with bladder cancer that contained 14 patients with low grade transitional cell carcinomas (LGTCC) and 26 patients with high grade transitional cell carcinomas (HGTCC) (Fig. 5a,b). In every instance, abnormal copy number levels were detected in touch-print preparations of the tumor tissue and the corresponding paired voided urine sample from the same patient. The percentage of cells with 3–4 copies and as well as percentage of cells with more than 4 copies of AURKA were similar in tumor and urine samples from the same patient (Fig. 5c). It was also evident that the proportion of cells with 3–4 and more than 4 copies of AURKA was higher in HGTCC than in LGTCC (Fig. 5d).

The performance of the FISH test for AURKA copy number was then evaluated in a blinded fashion in voided urine samples from several cohorts of patients and is summarized in Fig. 5e,f and Supplementary Fig. S5. Initially we tested the performance of AURKA FISH test in voided urine samples from 232 patients with bladder tumors and 255 control samples, 126 from healthy individuals and 129 from patients with benign urologic disorders. We used only the samples that contained at least 10 measureable cells, although in the majority of cases (90.6%) the number of analyzed cells was 20 or more, and by analyzing the ROC curve, we established a cutoff of positivity at greater than 20% of cells with at least three copies of AURKA. With these criteria, the AURKA FISH test was positive for samples from 185 patients with bladder cancer. Less than 20% abnormal cells with more than 2 AURKA gene copies were detected in samples from the remaining 47 patients with bladder cancer. In 24 of the 255 control samples, we detected more than 20% abnormal cells with more than two copies of the AURKA gene. Among the abnormal benign cases, one sample showed 10% of cells with more than 4 copies and three samples contained 10% of cells with more than 4 copies of AURKA. The percentage of control cases with cells having 3–4 copies of AURKA varied from 15 to 40%. ROC curve analysis of AURKA FISH data from the cohort yielded a specificity of 79.7% (95% confidence interval [CI] = 74.1% to 84.4%), a sensitivity of 79.6% (95% confidence interval [CI] = 74.2% to 84.1%), and an AUC of 0.901 (95% CI = 0.872 to 0.928; *P* < 0.001). (Figure 5e; Supplementary Fig. S4a,b). The degree of AURKA gene amplification, as measured by the AURKA score, was associated with the histological grade of the tumors. Bladder cancers of high histological grade (grade 3) had a significantly higher mean AURKA score than bladder cancers of low (grades 1–2) histological grade (0.5 vs 0.3, difference = 0.2, 95% CI = 0.10 to 0.39, *P* < 0.001) (Fig. 5f). To assess the predictive value of AURKA score we performed survival analyses. The cases were dichotomized into AURKA high and low scores by using the median value and the survival rates were analyzed in relation to grade and stage. As expected, the survival rates of low grade tumors were significantly better when compared to high grade tumors. The additional subdivisions of low and high grade tumors into those with low and high AURKA scores did not improve the prediction of survival (Fig. 5g). Similarly, the survival rates of patients with superficial tumors were significantly better when compared to the survival rates of patients with invasive tumors. The survival rates of patients with tumors showing low AURKA scores were somewhat better in both groups of patients i.e. those with superficial and invasive tumors but the differences were not statistically significant (Fig. 5h). The multivariate analysis using the Cox proportional hazard model disclosed that the AURKA score provides important and significant information on survival but adding the AURKA score to grade and stage does not improve the prediction of survival and therefore it is not an independent prognostic tool. To address the question whether AURKA FISH test can predict tumor recurrence we used 224 samples from patients with superficial bladder cancer that were followed for recurrence. The follow-up ranged in this cohort from 1 to 13 years with an average of 5.8 years. By using the same cutoff of positivity at greater than 20% of cells with at least three copies of AURKA, we did not see any difference between those cases which recurred as compared to those which did not (Supplementary Fig. S5c,d and e).

Since cytological examinations of cells exfoliated in voided urine is commonly used for non-invasive bladder cancer detection,^30^ we compared a selected number of cases of AURKA FISH test with cytology. The cytological analysis was performed on the samples with additional aliquots of urine sediments remaining on file after the completion of the AURKA FISH studies. It was performed in a blinded fashion and the cytopathologist was not aware of clinical and pathological diagnosis as well as the results of AURKA FISH tests. Cytology correctly identified 93 out of 144 samples as positive for bladder cancer (sensitivity = 64.6%, 95% CI = 56.6% to 72.6%), whereas the AURKA FISH test, using the same cut point for positivity of the test (ie, >20% of cells with abnormal AURKA gene copy number) correctly identified 132 samples as positive for bladder cancer (sensitivity = 91.7%, 95% CI = 87.1% to 96.3%) (Fig. 5i). Of the 51 samples that were classified as negative by cytology, 47 were correctly identified as positive by the AURKA FISH test. As a result we can state that the AURKA FISH test is significantly more sensitive than cytology.

## Discussion

Here we show that AURKA, a protein kinase best known for its role in promoting mitotic spindle assembly and mitosis, is overexpressed in basal bladder cancers and controls invasion in preclinical bladder cancer models. We identified the tumor suppressor gene NNMT as a downstream target of AURKA. In *in vitro* studies, up-regulation of AURKA inhibited NNMT expression, which in turn contributed to bladder cancer invasion. The effects of AURKA were executed in large part via PAX3-mediated suppression of NNMT. A study by Fang *et al*. has demonstrated that silencing of PAX3 downregulated AURKA which suggests that it is an upstream regulator of AURKA with a feedback mechanism^31^. The analysis of several non-overlapping cohorts of patients with bladder cancer showed that there is a subset of patients characterized by overexpression of AURKA and down-regulation of NNMT with poor clinical outcomes. This feature together with the expression signature of AURKA downstream regulatory targets was enriched in a highly aggressive basal intrinsic subtype of bladder cancer.

The ubiquitous involvement of AURKA in bladder cancer made it suitable to be an effective biomarker for its detection. Our FISH test for AURKA copy number in exfoliated urothelial cells from voided urine sediments detected bladder cancer with a high degree of specificity and sensitivity. Recent studies from other laboratories using real time PCR of voided urine samples showed that increased AURKA expression levels can be used for the detection of bladder cancer^32^. Moreover they emphasize that AURKA expression levels, as compared to urine cytology, are particularly effective for the detection of low-grade bladder cancers. In addition, these studies show that the levels of AURKA expression parallel the progression of the disease to higher stages^31^. AURKA score reflecting its degree of amplification shows strong correlation with high histologic grade and stage. A low degree of amplification is however present in low grade and superficial tumors making it an effective marker for this subset of bladder cancer. Although it appears that the AURKA score provides some prognostic information, it is not an independent predictor of survival in the multivariate Cox proportional hazard model. Comparison of AURKA FISH with a commonly used UroVysion test comprised of multicolor DNA probes for alpha satellite DNA mapping to 3p11.0-q11.1, 7p11.1-q11.1, 17p11.1-q11.1 and the p16 probe mapping to 9p21 suggests that AURKA may be a equally effective and less expensive alternative for non-invasive detection and monitoring of bladder cancer^33^.

This new role of AURKA in bladder cancer aggressiveness and its utility as a diagnostic marker for bladder cancers should be taken in the context of its known diverse functions with more than 60 interacting proteins^5^,^6^,^7^. AURKA localizes primarily in the centrosomes and regulates their function^6^. Amplification and overexpression of AURKA has been observed in many tumors, including bladder cancer, and it is believed to be contributory to aneuploidy and genomic instability^5^,^7^,^8^,^10^,^11^,^34^. The key functional partner proteins include MYCN, NFκB inhibitor α, AKT1, RALA, P53 and BRCA1^15^,^35^,^36^,^37^,^38^,^39^,^40^,^41^. AURKA modulates phosphorylation of these important oncogenic proteins resulting in their loss of function and contributing to upregulation of their respective pathways. Phosphorylation of P53 protein with its respective silencing is particularly relevant for bladder cancer as its loss of function represents the most frequent alteration found in the disease by genomic profiling^15^,^27^. The most recent identification of AURKA mediated phosphorylation of geminin causing the stabilization of the pre-replicative complex further expands the central role of this protein in regulating cell proliferation^41^,^42^. Previously identified AURKA dependent phosphorylation of RALA which stimulates cell migration is relevant to our observation as it may play a contributory role to tumor invasiveness^40^,^43^. Additional evidence for AURKA involvement in cell migration and invasion is provided by recent identification of its interactions with PLD, FAK and Src^43^.

Recently, several groups used whole-genome expression profiling to identify intrinsic subtypes of bladder cancer. In spite of identifying a different number of subclasses, all groups recognized that molecular subtypes of bladder cancer contain the so-called basal and luminal gene expression signatures that were surprisingly similar to those found in breast cancer^24^,^25^,^26^,^27^,^28^,^29^,^44^,^45^,^46^,^47^. In general, basal and luminal bladder cancers are distinguished by differential expression of genes associated with normal urothelial differentiation and show distinct expression of transcription factors and molecular pathways^24^,^25^,^26^,^27^,^28^,^29^,^44^,^45^,^46^,^47^. Most importantly they show significant differences in clinical behavior and sensitivities to frontline chemotherapy^24^,^25^. Specifically, basal cancers are characterized by expression of KRT5 and KRT14 and contain expression signatures controlled by active STAT3 and p63^24^,^25^. In contrast, luminal cancers express breast cancer differentiation markers (KRT20, CD24, ERBB2, and GATA3) and uroplakins which are markers of terminal urothelial differentiation. They also display gene expression signatures characteristic of active peroxisome proliferator activator gamma (PPARϒ) and are enriched for papillary histopathological features and the alterations of FGFR3^24^,^25^,^26^,^27^,^47^. The fact that AURKA and its downstream effects are highly enriched in basal cancers provides additional clues to the pathogenesis of this highly aggressive subtype of bladder cancer. It seems likely that AURKA overexpression and amplification are driver events in basal bladder cancer tumorigenesis, which could be readily tested in existing mouse models^46^,^48^.

Basal bladder cancers are associated with advanced stage i.e., extent of muscle invasion and metastatic disease at presentation, and they are enriched with biomarkers characteristic of epithelial-to-mesenchymal transition^24^,^25^, so our observation that AURKA is overexpressed in these tumors is entirely consistent with their other biological properties. Because AURKA amplification is readily detected in cells from urine sediments, the AURKA FISH test may prove useful in prognostication as a non-invasive marker for aggressive disease.

Our observation that NNMT inhibits invasion and metastasis in bladder cancer cells was completely unexpected. NNMT is a cytosolic enzyme that catalyzes the N-methylation of NCA, pyridines and analogues using S-adenosyl-L-methionine (AdoMet) as the methyl donor. Previous studies have examined the effects of NNMT knockdown or overexpression on various cancer cell phenotypes *in vitro*, yielding conflicting results^49^,^50^,^51^,^52^. Some of them identified positive correlation between overexpression of NNMT and tumor cell aggressiveness^50^,^51^. Others, similar to our observations, disclosed negative correlation, i.e. downregulation of NNMT resulted in an increased propensity for invasion and metastases and was associated with higher clinical aggressiveness^49^. For example, Sartini *et al*. have reported that NNMT upregulation inversely correlates with lymphnode metastasis in oral squamous cell carcinoma^53^. Similarly some studies found that overexpression of NNMT was associated with prolonged progression free and overall survival times^51^. Several additional studies found positive correlation between NNMT overexpression and invasion and metastasis in both *in vitro* and *in vivo* models of renal cell, pancreatic, and bladder cancers^54^,^55^,^56^. In view of these data, it appears that the effect of NNMT overexpression and downregulation is tissue and cancer specific. A recent study demonstrated that NNMT has global effects on chromatin structure (hypomethylation) by virtue of its ability to deplete cellular pools of S-adenosyl methionine^57^,^58^. Chromatin-modifying enzymes are frequently mutated in invasive bladder cancer^27^,^59^, consistent with the idea that chromatin modifications play critical roles in bladder cancer progression. We should emphasize that although there is enrichment of the AURKA signature comprising its downstream target genes in basal bladder cancer not all AURKA overexpressing tumors show downregulation of NNMT. This indicates that other AURKA related mechanisms may be operating in such instances.

In summary, our study has identified a novel mechanism for AURKA regulating cell invasion and contributing to aggressive clinical behavior of bladder cancer and has established a role for AURKA as a detection marker for the disease. The results of our study have important implications for diagnosis, management and treatment of bladder cancer.

## Methods

### Patients and tissue samples

All human tissues used in this study were collected under protocols reviewed and approved by the Institutional Review Board of The University of Texas MD Anderson Cancer Center and collaborating institutions. The informed consents were obtained from all subjects who provided tissue samples and urine for this study. All studies were performed in accordance with the relevant guidelines and regulations. Clinical and pathological data on 1,404 patient samples organized into several study cohorts are summarized in Supplementary Table S2. The expression levels of AURKA and NNMT were evaluated by tissue microarray comprising formalin-fixed and paraffin-embedded (FFPE) bladder tumor samples from 423 patients (325 men and 98 women) and annotated follow-up data. These included 57 LGTCC, and 366 HGTCC together with four sections of normal human ureters from nephrectomy specimens.

Additional validations were performed on the two genomically profiled cohorts of bladder cancer sample sets. The first cohort (TCGA) represented sample set of fresh frozen 128 high grade, muscle invasive (T_2_ and higher), bladder tumor samples. The second cohort (MDA) consisted of 142 fresh frozen bladder tumor samples from 105 men and 37 women. These included 45 low-grade and 97 high-grade tumors comprising 60 superficial papillary urothelial carcinomas and 82 invasive non-papillary carcinomas. A set of eight normal human urothelial cell samples from nephrectomy specimens without urothelial carcinoma served as controls. Both cohorts were genomically profiled, as previously described^25^,^27^.

We used 711 voided urine samples from 590 subjects to assess the performance of AURKA FISH test for the detection and monitoring of bladder cancer. The voided urine samples were dichotomized for a control set comprising 255 samples (126 samples from normal subjects and 129 samples from patients with benign urologic disorders) collected at The University of Texas Southwestern Medical Center and 232 samples from patients with Bladder Cancer collected at The University of Texas MD Anderson Cancer Center. In addition, 224 samples from patients with a history of bladder cancer collected at The University of Texas MD Anderson Cancer Center and Baylor College of Medicine were used to assess the ability of AURKA FISH test to predict tumor recurrence.

Transitional cell carcinomas (TCC) were classified according to the histologic tumor grading system of the World Health Organization^60^ and were dichotomized as low-grade or high-grade tumors. The growth pattern of papillary versus non-papillary or solid tumors and the depth of invasion were also recorded. Levels of invasion were defined according to the TNM staging system^61^. T_1_ tumors were substaged as T_1a_ or T_1b_ to dichotomize them as superficial (T_a_ − T_1a_) or invasive (T_1b_ and higher) as previously described^62^.

### Cell lines

*In vitro* studies of AURKA biologic effects were performed on immortalized normal human urothelial cells (NHU) and UC5, UC6, UC7, UC9, UC10 and UC11 bladder cancer cell lines which were cultured as previously described^63^.

### Modulation of AURKA and NNMT by shRNA

Silencing of AURKA and NNMT was performed by stable transfection with lentiviral shRNA containing the following shRNA sequences: AURKA-pLV-F: 5′-CGCGTCCCCTGGTAAAGCTGTTGGAATGTTCAAGAGACATTCCAACAGCTTTACCATTTTTGGAAAT-3′ and AURKA-pLV-R: 5′-CGATTTCCAAAAATGGTAAAGCTGTTGGAATGTCTCTTGAACATTCCAACAGCTTTACCAGGGGA-3′; NNMT-pSIH-F: 5′-GATCCGAAAGAGGCTGGCTACACACTTCCTGTCAGATGTGTAGCCAGCCTCTTTCTTTTTG-3′ and NNMT-pSIH-R: 5′-AATTCAAAAAGAAAGAGGCTGGCTACACATCTGACAGGAAGTGTGTAGCCAGCCTCTTTCG-3′). The following non-targetable shRNA sequence was used as control: 5′-TTCTCCGAACGTGTCACGT-3′. The lentivirus system and cell transduction were generated as described previously^64^.

In order to rescue AURKA expression in stably silenced cells, we introduced five silent point mutations into the expression vector at the region targeted by the AURKA shRNA, using the QuikChange II XL site-directed mutagenesis kit (Stratagene). The rescue AURKA vector was stably transfected into the AURKA-silenced cells. The same cells transfected with an empty vector served as control.

### Methyltransferase activity of NNMT

To analyze the methyltransferase activity of NNMT, we used an assay based on the conversion of nicotinamide to radioactively labeled N-methylnicotinamide with ^14^C-Ado-Met as the methyl donor as previously described^65^. Cell lysates were prepared from UC7 and UC11 cell lines with shRNA silenced AURKA and NNMT were incubated with Nicotinamide and ^14^C-Ado-Met. Lysates incubated with DMSO served as controls. The radioactivity was measured using a Beckman liquid scintillation counter.

### MTT proliferation assay

Ninety-six-well plates containing 2500 cells/well from UC7 and UC11 cell lines of silencing AURKA, UC5 with overexpression of AURKA, and control were cultured for 5 days and analyzed by 3-(4,5-dimethylthiazolyl-2)-2,5-diphenyltetrazolium bromide (MTT) assay, which determines relative cell numbers based on the conversion of MTT to formazan in viable cells. MTT (60 μg/ml) was added to each well and incubated for 2 hours. The medium was removed and 100 μl of dimethyl sulfoxide was added to lyse cells and solubilize formazan. Absorbance was determined on a microplate reader.

### Transcriptional regulation of NNMT by PAX3

Transcriptional regulation of NNMT by PAX3 was tested by reporter gene constructs and luciferase activity analysis as previously described^66^. The NNMT promoter was cloned from UC11 cells to encompass 600 bp upstream of the transcription initiation site. The transcription factor binding sites in the NNMT promoter were identified by the Genomatix.de program. This program identified several transcription binding sites for STAT-3, HNF-1, CREB, ATF-1 and PAX-3 upstream of NNMT. We decided to concentrate on PAX-3′s two binding sites located within 700 bp from the transcription initiation site because previous studies demonstrated that it inhibited the transcription of NNMT. Direct site mutagenesis of PAX3 binding sites was carried out using Quikchange II XL Site Directed Mutagenesis Kit (Stratagene) according to manufacturer’s protocol and luciferase reporter plasmids were transfected into the non-targeting (NT) cells.

### Chromatin immunoprecipitation (Chip) assay

PAX3 binding to the promoter region of NNMT was tested by chromatin immunoprecipitation assay carried out by using the ChIP-IT Express Kit from Active Motif^64^. Protein–DNA complexes were pulled down with anti–PAX3 antibody (Santa Cruz). PCR was performed using the following primers; forward 5′-CCAACATTCCTTAGCCCTGA-3′ and reverse 5′-AGACCAGAGGGAGCACTTGA-3′.

### Expression and activity of MMPs

The expression levels of MMP2 protein were tested in UC7 and UC11 cells by ELISA per the manufacturer’s protocol. The total MMP2 levels were assessed in the supernatants using Quantikine Immunoassay kits (R&D Systems). The activity of MMP2 was measured by zymography assay (Biorad) as described previously^67^.

### *In vitro* cell invasion assays

The effects of AURKA and NNMT on cell invasion *in vitro* were carried out by Matrigel invasion assay using BioCoat Matrigel invasion chambers (BD Biosciences) as described previously^64^.

### Western blot analyses

To detect the expression of AURKA, NNMT, PAX3, and MMP2/9 20 μg of protein lysates were loaded on SDS-PAGE and western blots were performed as described previously^64^. Blots were incubated with primary antibodies (1:1000, anti-AURKA; Biolegend,1:1000, anti-NNMT; Genway Bio,1:1,000, anti-MMP2, anti-MMP9 and anti-PAX3; Cell Signaling Technology).

### Expressions of AURKA and NNMT in tissue microarrays

The expression levels of AURKA and NNMT were tested on tissue microarray comprising 423 formalin-fixed and paraffin-embedded bladder tumor samples designed and prepared as previously described^67^. Sections of four normal ureters, from nephrectomy specimens with no evidence of urothelial neoplasia, were included in the microarray and served as internal positive controls. Immunohistochemical staining for AURKA (Gene Tex GTX21287) and NNMT (Abcam, ab119758) was performed according to a standard immunohistochemical protocol. The proportions of tumor cells staining positive for immunohistochemically visualized antigens were measured using an automated digital image analyzer, GenoMx (BioGenex).

### Microarray experiments and data processing

Microarray experiments and data processing were performed as previously described^25^. Total RNA from fresh frozen cells was isolated using the mirVanna™miRNA isolation kit (Ambion, Inc.) and direct hybridization assays were performed using the Illumina RNA amplification kit (Ambion, Inc.) and Illumina HT12 V3 chips (Illumina, Inc.). Slides were scanned with Bead Station 500X and the significantly differentially expressed genes for each comparison (P < 0.05 with FDR <0.1, ≥1.5 fold change) were extracted, analyzed and displayed as heatmaps.

### Expression of AURKA, PAX3, NNMT and MMP2/9 in molecular subtypes of bladder cancer

The analyses of expression levels of AURKA, NNMT and MMP2/9 as well as the downstream AURKA target genes in molecular subtypes of bladder cancer were performed on two genomically profiled bladder cancer cohorts. The RNAseq data for the TCGA cohort and gene expression profile obtained from RNA isolated from tumor samples of the MDA cohort using Illumina HumanHT12 V3 v chips were analyzed using previously described algorithms^25^. The tumors were classified into two major molecular subtypes as luminal and basal. A subtype of luminal cancers characterized by the upregulation of p53 downstream regulatory genes was also identified. Gene set enrichment analyses of AURKA downstream target genes, defined by the literature, were tested for enrichment in luminal and basal tumors. In addition, copy number data of the TCGA cohort were downloaded and the segmentation data which were processed by the TCGA consortium and open to the public were used to generate the heatmaps of copy number data in molecular subtypes of bladder cancer.

### Urine samples analyzed by FISH

Voided urine specimens (approximately 100–200 mL) were collected and prepared for FISH analysis as previously described^19^. Urine sediments from patients with bladder cancer were used for cytological analysis by staining with the Papanicolaou technique to compare the performance of cytology and AURKA FISH test. The cytological analysis was performed on 144 samples with additional aliquots of urine sediments remaining on file after the completion of AURKA FISH. Slides from frozen tumor tissue were prepared by touch print as previously described. Dual color AURKA (20q13)/20q11 probe (Kreatech, Durham, NC) was used to test the AURKA gene copy number.

### General analysis of data

The statistical difference between the sample sets was tested by using unpaired t-tests. Association between survival and tumor phenotypes were assessed by using Kaplan Meyer curves and the significance was tested by the Wald test.

## Additional Information

**How to cite this article**: Mobley, A. *et al*. Aurora Kinase A is a Biomarker for Bladder Cancer Detection and Contributes to its Aggressive Behavior. *Sci. Rep.* **7**, 40714; doi: 10.1038/srep40714 (2017).

**Publisher's note:** Springer Nature remains neutral with regard to jurisdictional claims in published maps and institutional affiliations.

## Supplementary Material

Supplementary Information
