# Supplementary material for: Aurora Kinase A is a Biomarker for Bladder Cancer Detection and Contributes to its Aggressive Behavior
Source: Sci Rep. 2017 Jan 19;7:40714. doi: 10.1038/srep40714 (PMC5244380; doi:10.1038/srep40714)
Supplement: Supplementary Information [file srep40714-s1.pdf]

Aurora Kinase A is a Biomarker for Bladder Cancer Detection and Contributes to its Aggressive Behavior

Aaron Mobley[1†], Shizhen Zhang[2†], Jolanta Bondaruk[2†], Yan Wang[2], Tadeusz Majewski[2], Nancy P. Caraway[2], Li Huang[1], Einav Shoshan[1], Guermarie Velazquez-Torres[1], Nitti Giovanni[1], Sangkyou Lee[2], June Goo Lee[2], Enrique Fuentes-Mattei[2], Daniel Willis[4], Li Zhang[3], Charles C. Guo[1], Hui Yao[3], Keith Baggerly[3], Yair Lotan[5], Seth P. Lerner[6], Colin Dinney[4], David McConkey[4], Menashe Bar-Eli[1*] and Bogdan Czerniak[2*]

Legends for Supplementary Figures

Supplementary Figure S1. Growth rate of bladder cancer cell lines after silencing and overexpression of ARUKA assessed by MTT assay. There is no significant change in growth rate of UC7 and UC11 cells before and after silencing AURKA and in UC5 cells after overexpression of AURKA.

Supplementary Figure S2. Tissue microarray analysis of AURKA and NNMT expressions. (**a**) Quantitative analysis of AURKA and NNMT expressions in tissue microarray comprising 423 formalin-fixed and paraffin embedded bladder tumor samples as well as four sections of normal human ureters. The results are presented as a heatmap of expression levels of AURKA and NNMT in superficial (Ta) tumors and three different groups of invasive (T1 and higher) bladder cancers as follows: GA, Low expression of AURKA and NNMT; GB, High expression of AURKA and NNMT; and GC, High expression of AURKA and low expression of NNMT. (**b**) Dot plot demonstrating expression of AURKA and NNMT in superficial (Ta) tumors and in three groups of invasive (T1 and higher) bladder cancers generated using the following percentage of positive tissue staining cutoffs: 10% for AURKA and 50% for NNMT. (**c**) Percentage of LGTCC and HGTCC in superficial (Ta) bladder tumor samples and in the three groups of invasive (T1 and higher) bladder tumor samples defined as GA, GB, and GC described above.

Supplementary Figure S3. Expression of AURKA, NNMT, and MMP2/9 in the MDA cohort of bladder tumor samples.

(**a**) Expression pattern of AURKA, NNMT and MMP2/9 as well as AURKA signature genes in molecular subtypes of bladder cancer in the MDA bladder cancer cohort comprising of 142 fresh frozen bladder tumor samples with follow-up data. The samples were classified into luminal, p53-like, and basal subtypes using the previously published algorithm. Subsets of tumors with overexpression of ARUKA (ARUKA-H) and subsets of tumors showing overexpression of

ARUKA (ARUKA-H) with downregulation of NNMT in each molecular subtype are shown. (**b**) Expression levels of ARUKA and NNMT in superficial (Ta) tumors and luminal and basal subtypes of invasive (T1 and higher) bladder cancers. (**c**) Proportion of tumors with overexpression of ARUKA (AURKA-H) and proportion of tumors which overexpress ARUKA (ARUKA-H) with downregulation of NNMT (NNMT-L) are shown. (**d**) Enrichment analysis of AURKA down-stream regulatory pathway genes in basal as compared to luminal forms of bladder cancer revealed by GSEA. The expression level of top 10 up-regulated and down-regulated genes is shown. (**e**) Kaplan-Meier analyses of disease-specific survivals in molecular subtypes of bladder cancer and in relation to AURKA and NNMT expression levels. Superficial (Ta) tumors were separated from invasive (T1 and higher) samples.

Supplementary Figure S4. Genome-wide copy number variations in molecular subtypes of bladder cancer in the TCGA cohort. (**a**) Heatmap depicting copy number variations in individual cases segregated according to their molecular subtypes. Individual panels correspond to chromosomes 1-22 and X indicated on the left side of the diagram. Rows correspond to individual tumor samples. (**b**) Fraction of genome exhibiting copy number variations (CNV) in individual cases classified according to their molecular subtype. (**c**) Mean fraction of genome with copy number variations in three molecular subtypes of bladder cancer. (**d**) Mean fraction of genome with copy number variations in bladder cancer cases with high (H) and low (L) levels of AURKA expression. (**e**) Relationship between AURKA expression and fraction of genome with copy number variations (CNV). Corr; Pearson correlation coefficient.

Supplementary Figure S5. The results of AURKA FISH tests in several patient cohorts. (**a**) Summary of AURKA FISH test in 255 control voided urine samples from healthy individuals and patients with non-neoplastic disorders of the urinary tract. (**b**) Summary of AURKA FISH

test results performed on 232 samples of voided urine from patients with various pathogenetic subsets and stages of bladder cancer. (**c**) Summary of AURKA FISH tests in 224 patients with bladder cancer followed for recurrence. (**d**) Average proportion of cells with AURKA abnormal copy numbers in patients followed for recurrence. (**e**) Percentage of samples with positive AURKA FISH test in patients followed for recurrence. (**f**) Age distribution patterns of four distinct cohorts of patients whose voided urine samples were tested by AURKA FISH tests. *Inset* shows the distribution of bladder cancer patients by histologic stage and .grade.

Supplementary Figure S6. Full length blots for Figure 1a, 1b, 1d, 1g, and 1k.

Supplementary Figure S7. Full length blots for Figure 2b and full length gels for Figure 2d.

Supplementary Figure S8. Full length blots for Figure 3a and full length zymograms for Figure 3b.

Supplementary Figure 1

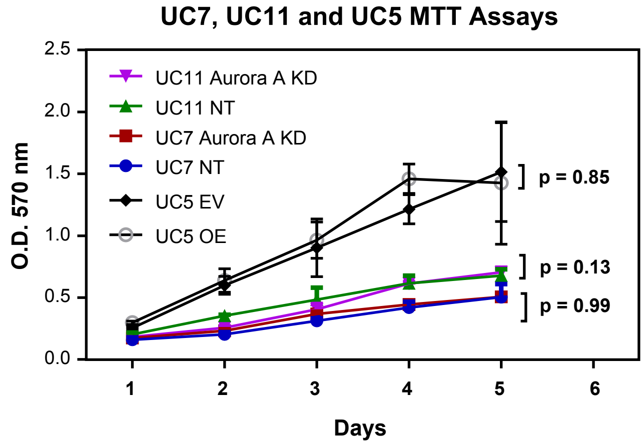

Supplementary Figure 2

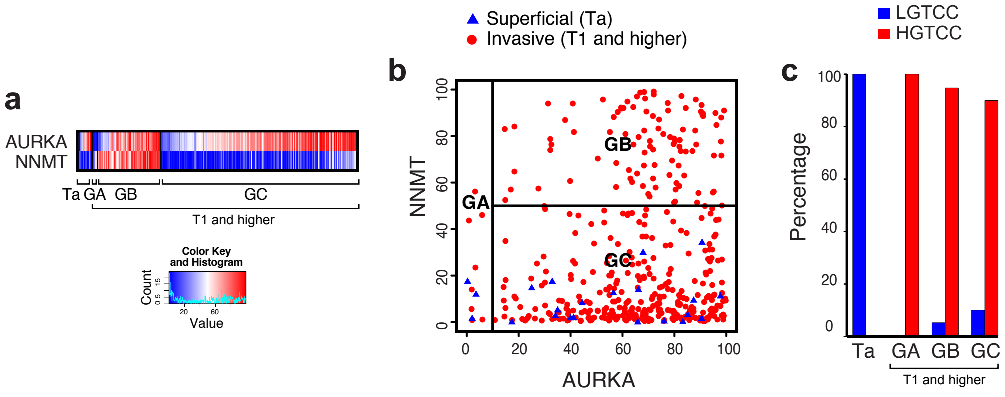

Supplementary Figure 3

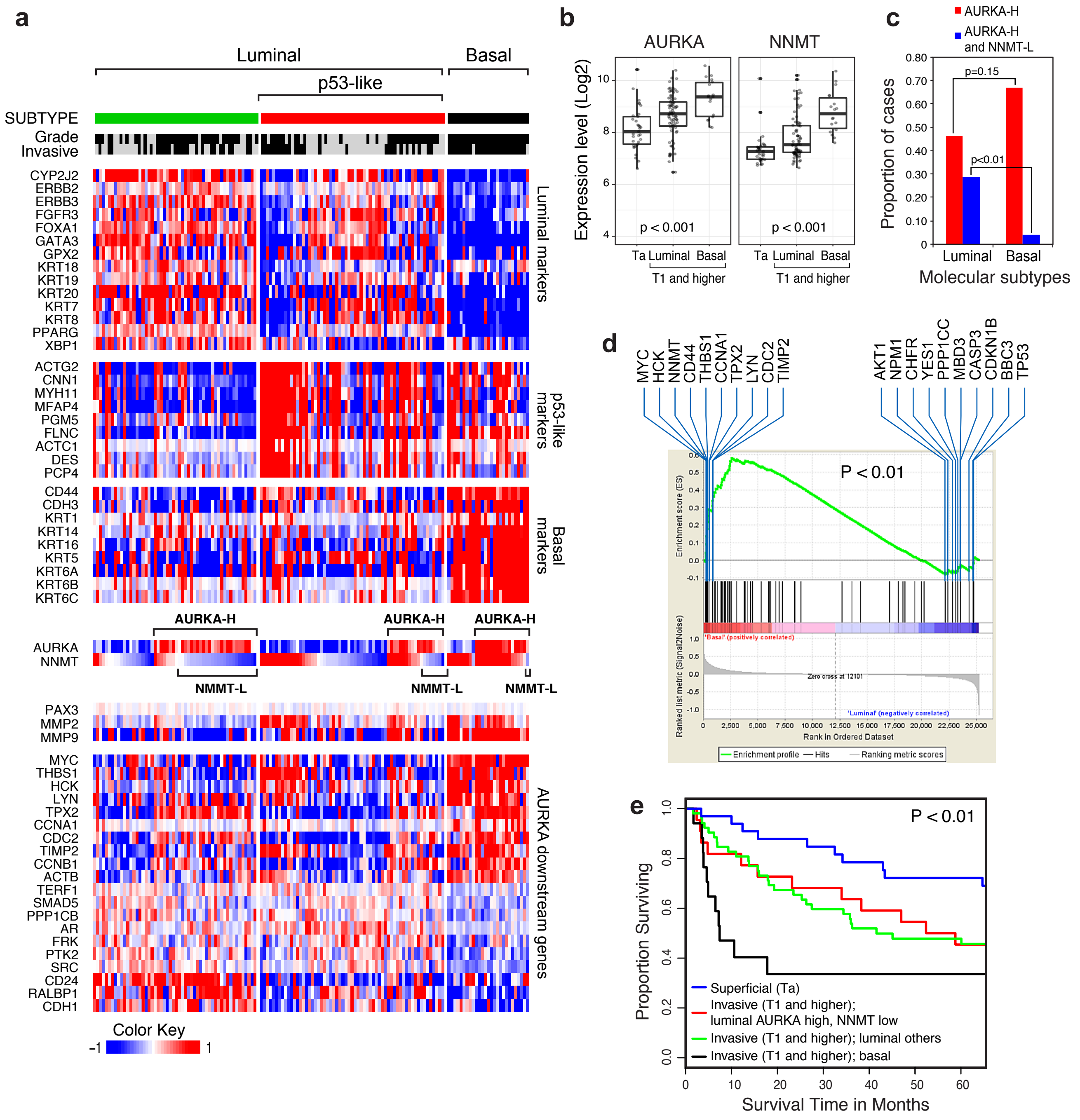

Supplementary Figure 4

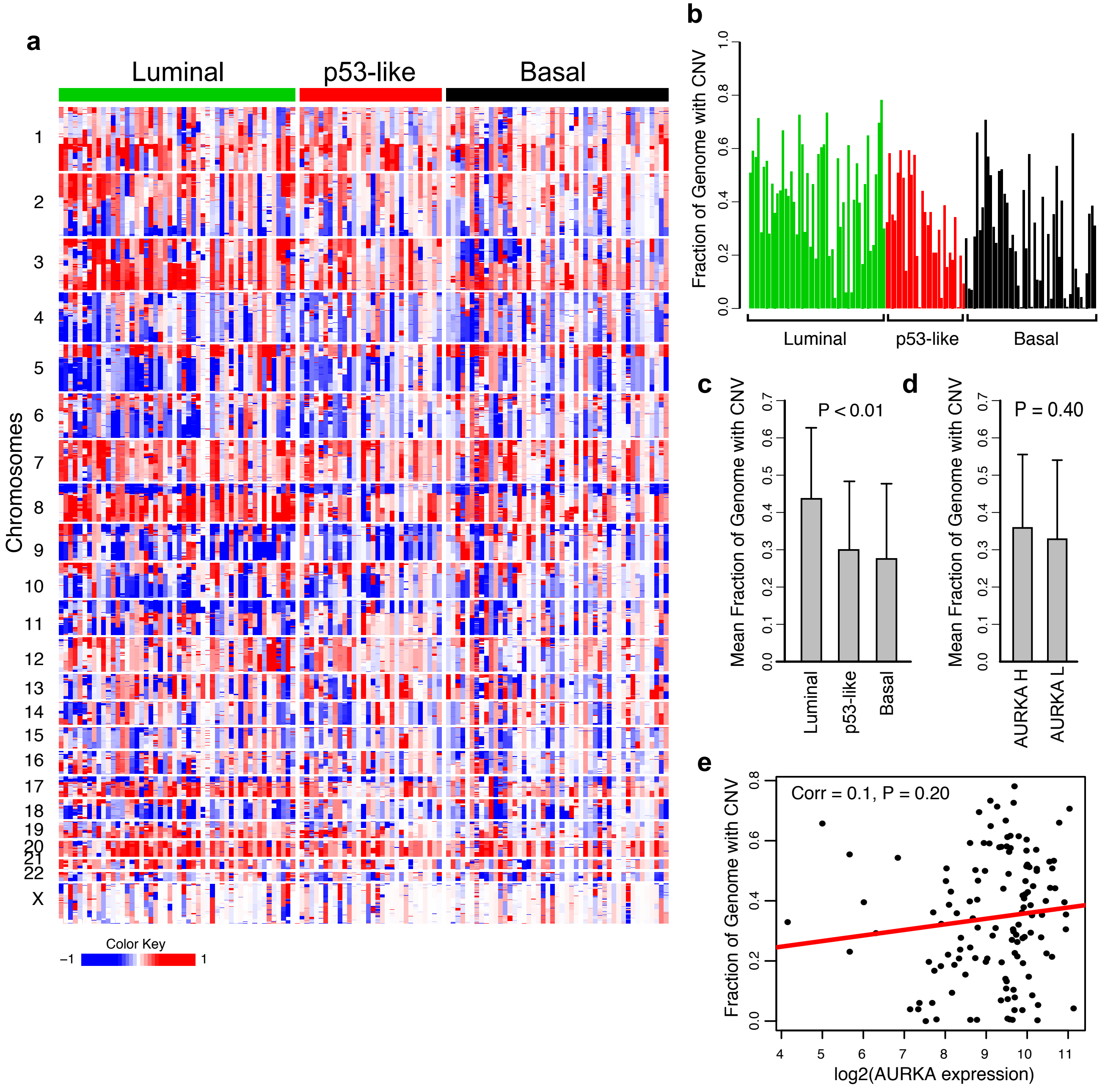

Supplementary Figure 5

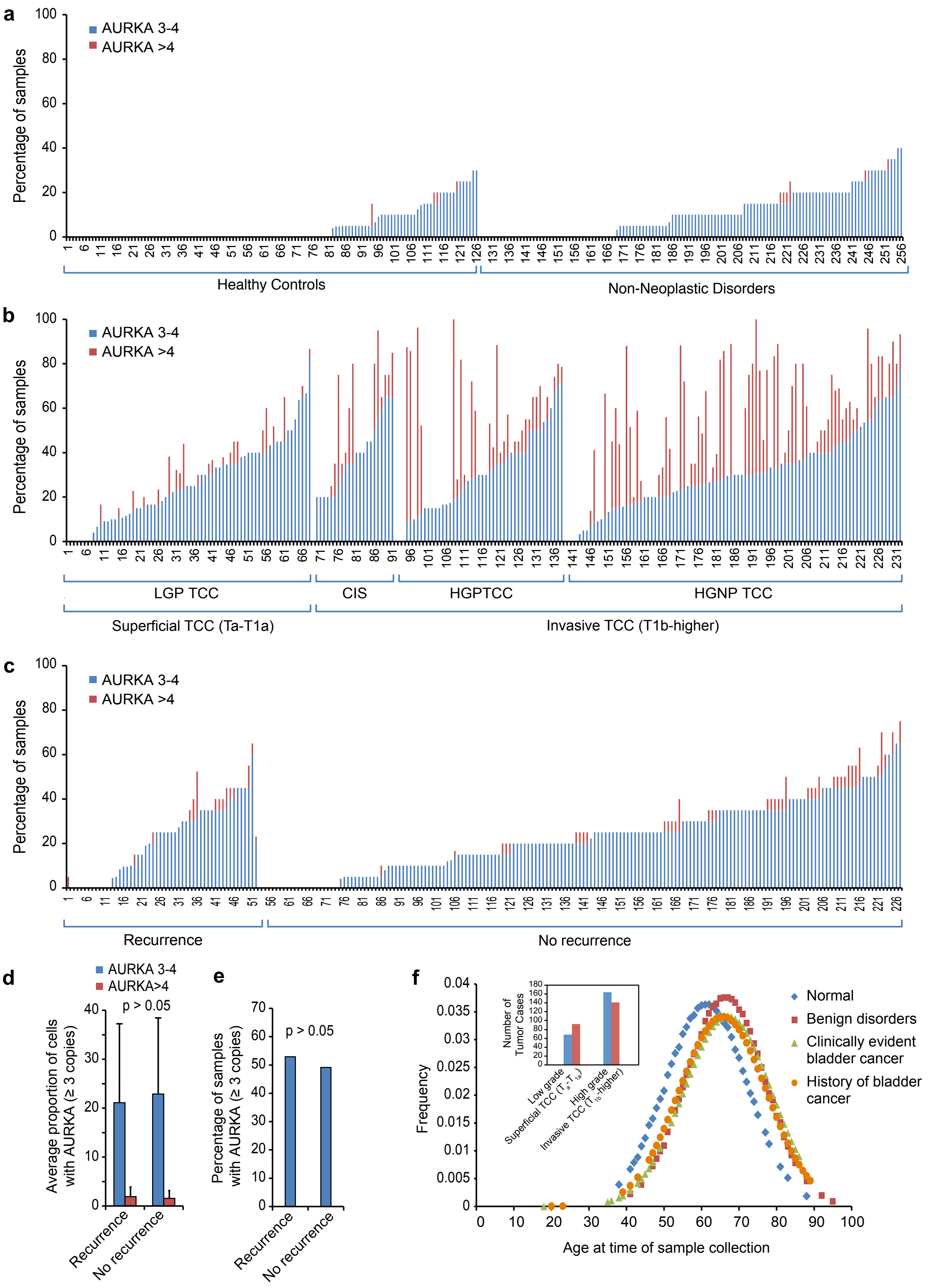

Supplementary Figure 6

Full length blots for Figure 1a

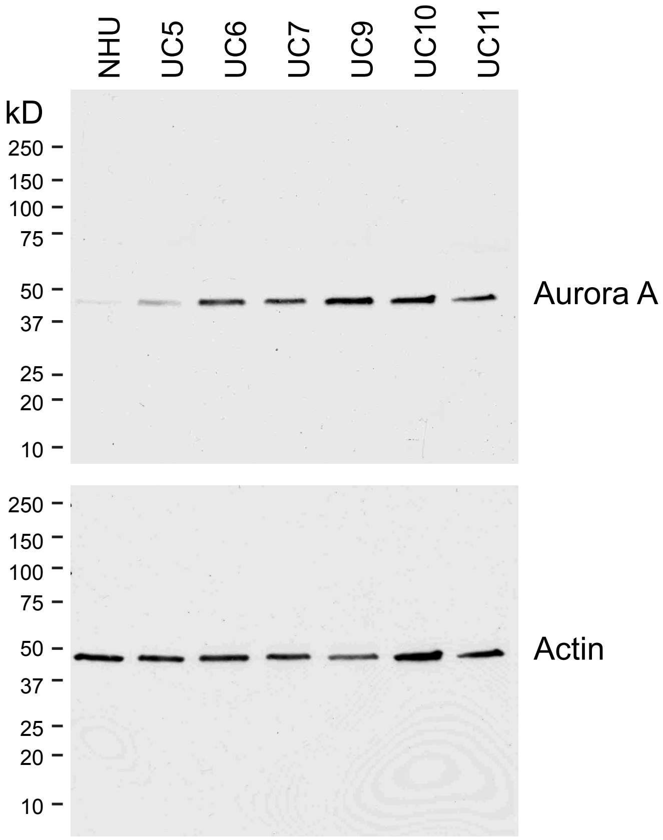

Full length blots for Figure 1b

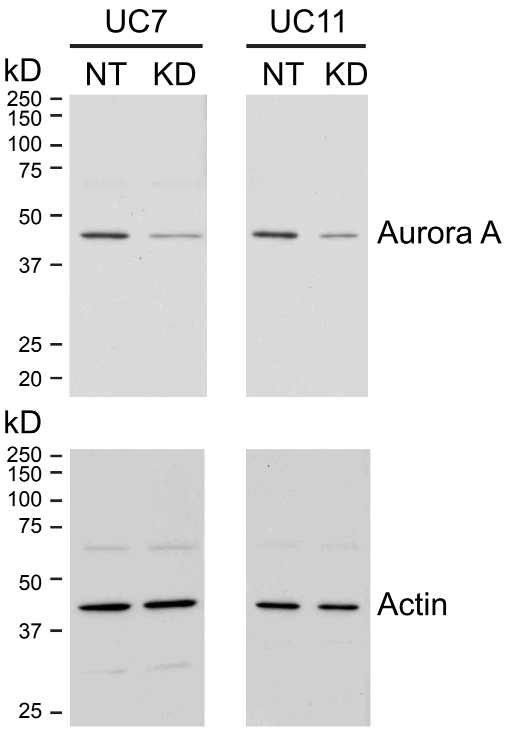

Full length blots for Figure 1d

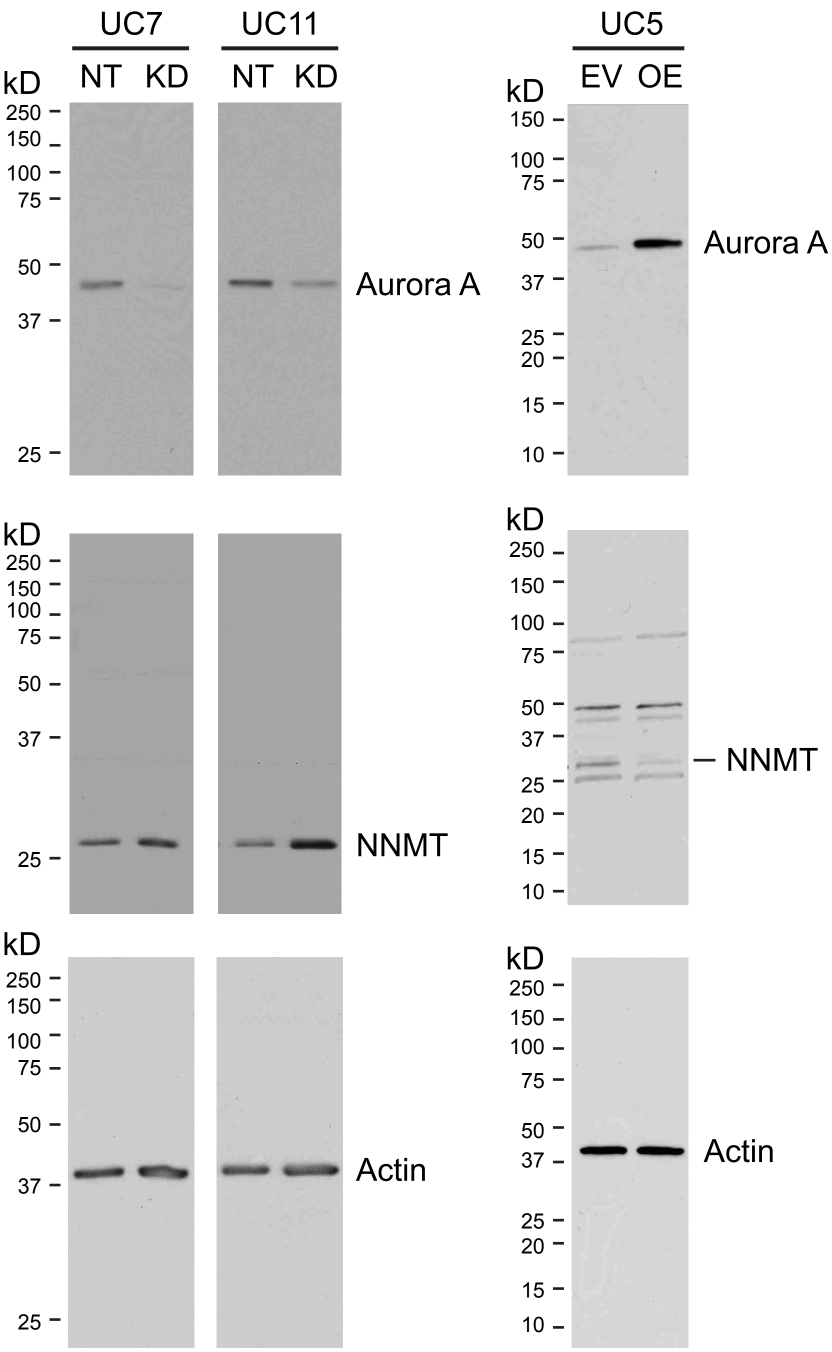

Full length blots for Figure 1g

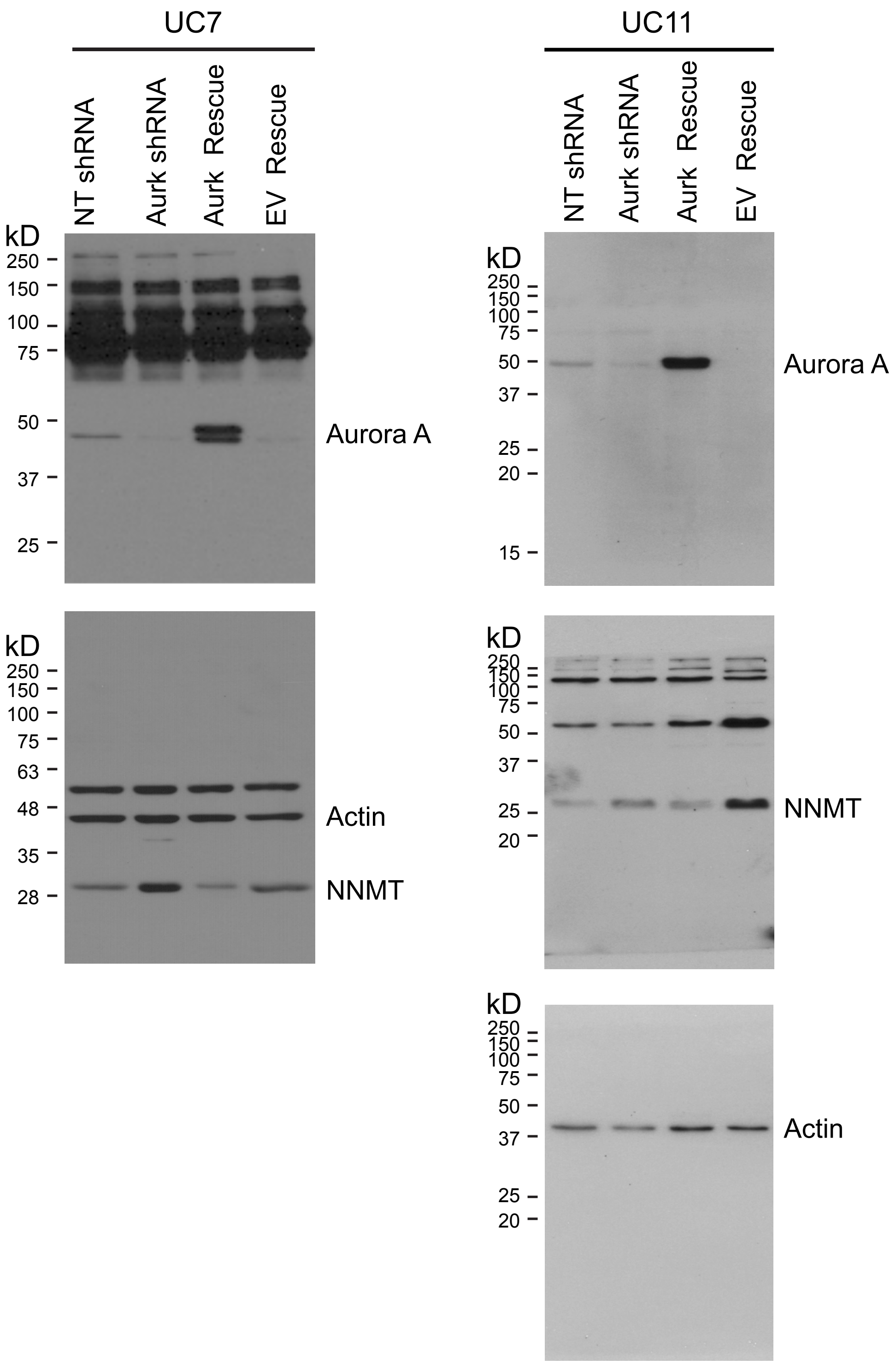

Full length blots for Figure 1k

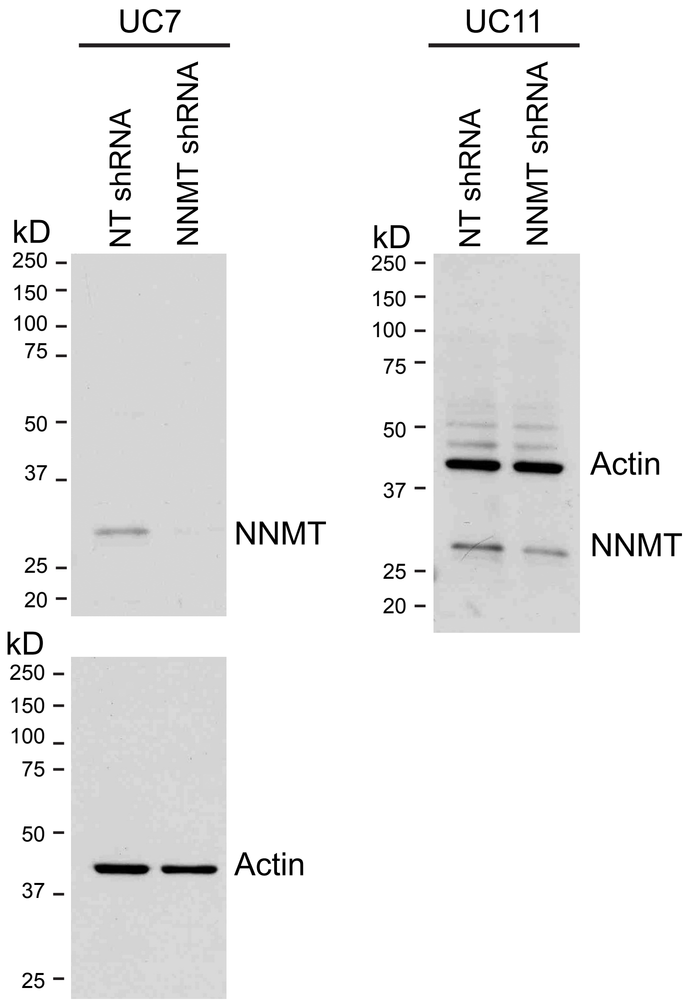

Supplementary Figure 7

Full length blots for Figure 2b

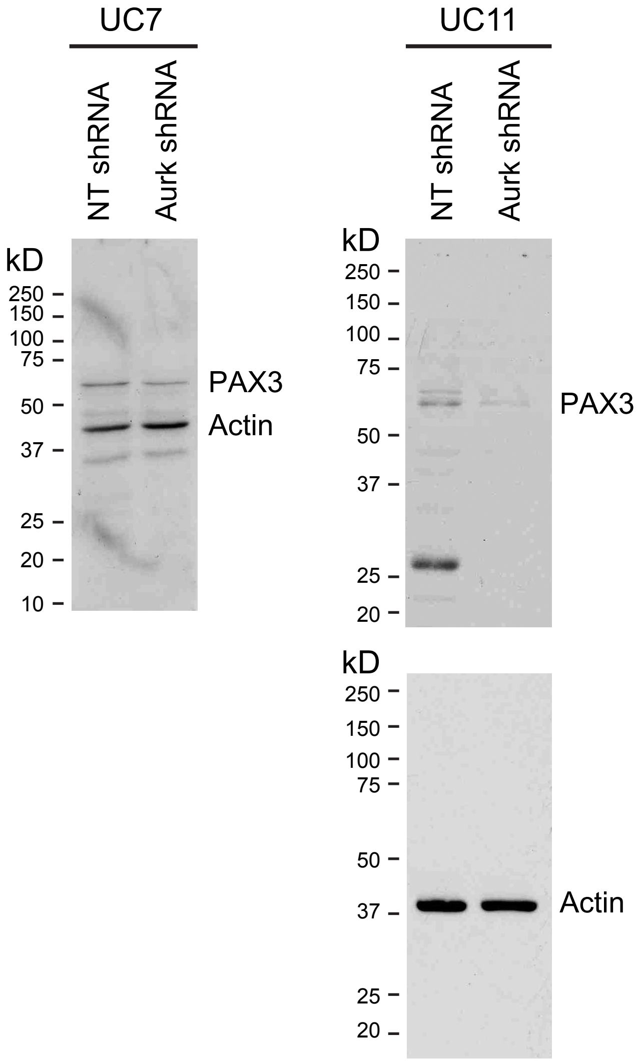

Full length gels for Figure 2d

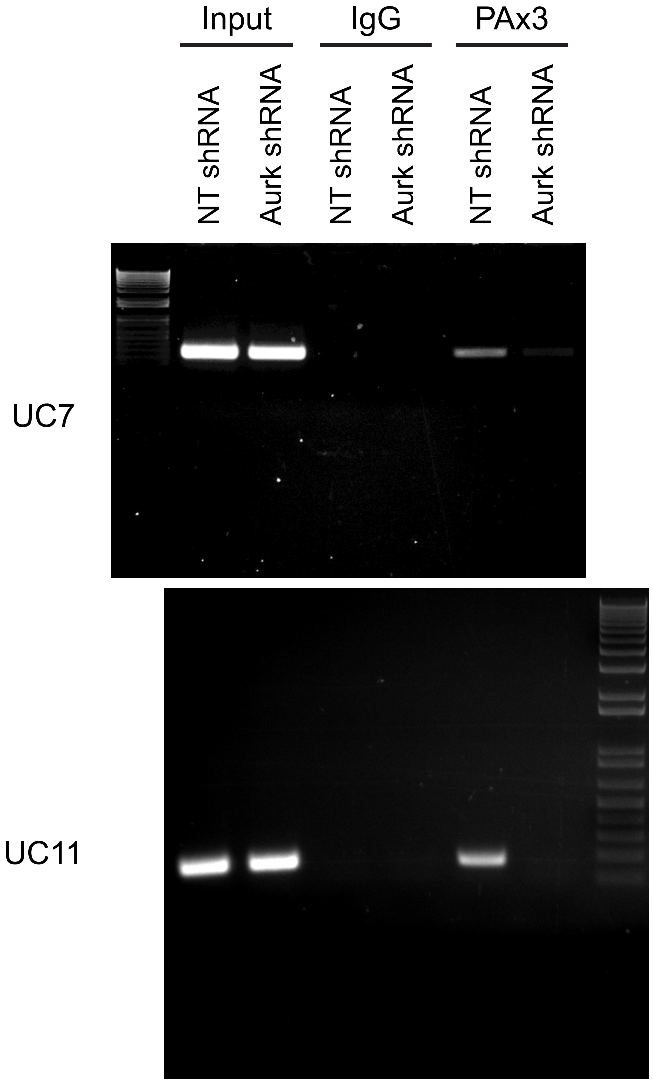

Supplementary Figure 8

Full length blots for Figure 3a

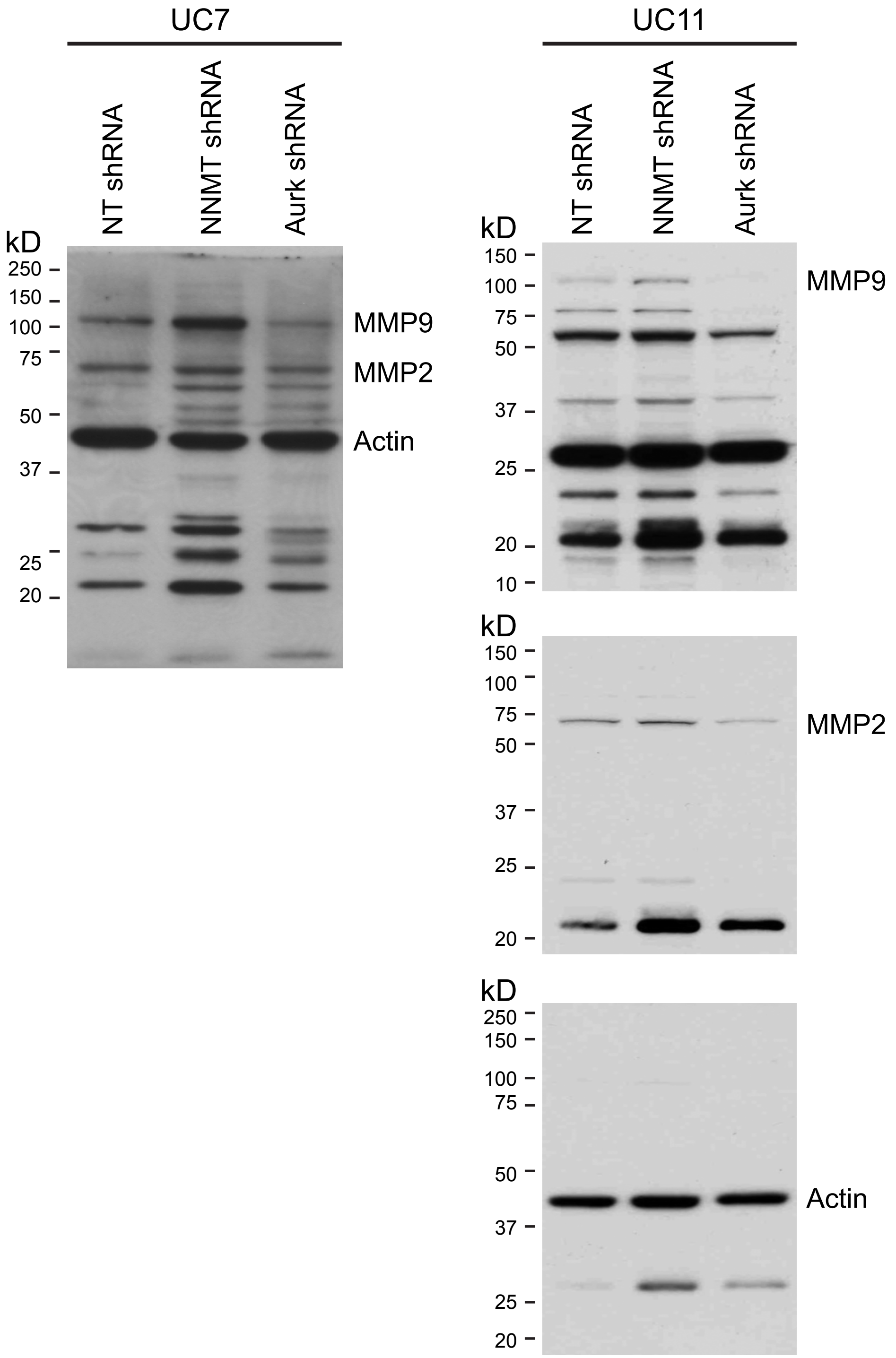

Full length zymograms for Figure 3b

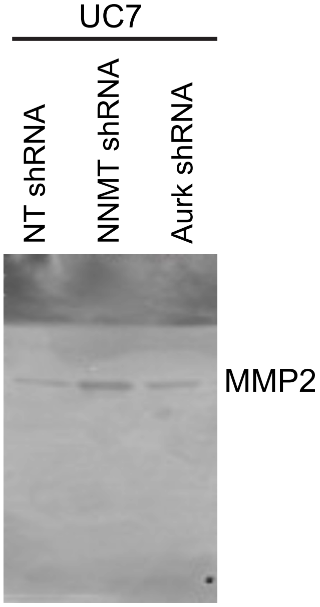

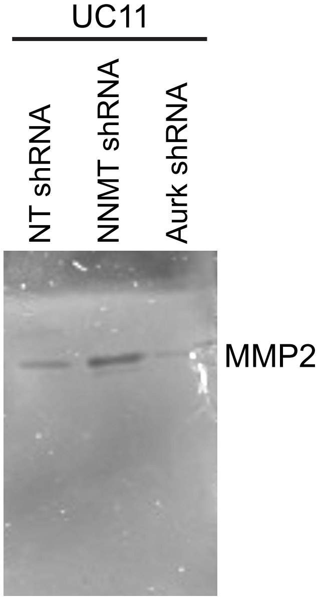

Supplementary Table 1. Expression levels of 10 most significantly up- and downregulated genes after silencing after AURKA*

Gene Symbol	UC11EV1	UC11EV2	UC11EV3	UC11shRNA1	UC11shRNA2	UC11shRNA3
AURKA	0.4	0.6	0.4	-0.4	-0.7	-0.4
MYEOV	0.6	0.5	0.4	-0.4	-0.6	-0.4
IL24	0.6	0.2	0.2	-0.2	-0.4	-0.5
CLPTM1	0.4	0.3	0.4	-0.3	-0.3	-0.3
GNB4	0.4	0.4	0.1	-0.1	-0.3	-0.5
LOC100190938	0.5	0.2	0.2	-0.3	-0.5	-0.2
STAG3L3	0.5	0.4	0.1	-0.3	-0.4	-0.1
FKBP14	0.5	0.3	0.2	-0.2	-0.5	-0.2
CHRNA5	0.5	0.2	0.2	-0.2	-0.4	-0.2
RFX1	0.4	0.3	0.3	-0.3	-0.3	-0.3
MGP	-0.3	-0.4	-0.7	0.7	0.5	0.3
NNMT	-0.4	-0.4	-0.4	0.6	0.4	0.5
MMP7	-0.3	-0.6	-0.6	0.4	0.3	0.4
C1S	-0.3	-0.3	-0.5	0.5	0.3	0.6
PLOD2	-0.4	-0.3	-0.6	0.4	0.4	0.3
F2RL2	-0.4	-0.4	-0.4	0.5	0.4	0.5
ITGAV	-0.4	-0.2	-0.8	0.3	0.2	0.4
TGFB2	-0.5	-0.3	-0.4	0.6	0.3	0.4
SNORA18	-0.5	-0.3	-0.5	0.6	0.3	0.3
GOLPH4	-0.5	-0.1	-0.5	0.1	0.4	0.7

UC11EV1-3 control samples transduced with non-targeting lentiviral vector.
UC11shRNA1-3 samples transduced with shRNA AURKA expressing lentiviral vector.
*The values represent the ranking of significantly upregulated and downregulated genes according to the average log-fold change. The significant genes were identified by the t-test (p value <0.01).

Supplementary Table 2. Summary of clinical and pathologic data of human samples (n=1404)

	Number	Gender		
	of samples	F	M	Mean Age±STDEV
TISSUE SAMPLES				
TMA	423	98	325	66.94±10.40
LGTCC*	57	12	45	65.79±10.31
HGTCC**	366	86	280	66.98±10.38
cDNA Microarray				
MDA Cohort	142	37	105	67.20±12.30
LGTCC	45	10	35	66.69±10.15
HGTCC	97	27	70	67.38±13.09
TCGA Cohort	128			
HGTCC	128			
URINE SAMPLES				
Control Samples	255	116	139	63.73±11.06
Healthy individuals	126	77	49	61.18±10.98
Benign disorders	129	39	90	66.23±10.60
Benign prostatic hyperplasia	40	0	40	69.93±7.72
Dysfunctional Bladder	28	18	10	59.88±9.47
Hematuria	12	7	5	62.42±9.96
Elevated PSA	11	0	11	64.25±11.14
Hyperlipidemia/Hypercholesterolemia	7	2	5	64.48±7.62
Kidney stone	5	1	4	58.40±7.12
Renal failure	3	1	2	62.00±12.53
Stricture of Ureter	3	1	2	50.67±3.51
Neurogenic bladder	2	2	0	55.50±20.51
Cystitis	2	1	1	68.50±6.36
Others	16	6	10	68.59±10.01
Tumor Samples	232	44	188	66.93±11.65
LGTCC	68	20	48	65.91±12.79
CIS***	22	4	18	64.05±11.06
HGP TCC	142	20	122	67.85±11.21
Superficial TCC (Ta-T1a)	99	24	75	64.93±12.54
Invasive TCC (T1b-higher)	133	20	113	68.41±10.75
History Samples	224	61	163	65.63±11.70
Total	1,404			

*LGTCC - low grade transitional cell carcinoma; **HGTCC - high grade transitional cell carcinoma; ***CIS - carcinoma in situ
